# The Influence of Lifestyle on Academic Performance Among Health Profession Students at Umm Al-Qura University

**DOI:** 10.7759/cureus.56759

**Published:** 2024-03-23

**Authors:** Huda A Mahfouz, Nawaf F Alhazmi, Maha K Almatrafi, Suhaylah S Almehmadi, Jehad K Alharbi, Lyan R Qadi, Abdullah Tawakul

**Affiliations:** 1 College of Medicine, Umm Al-Qura University, Al-Qunfudah, SAU; 2 College of Medicine, Umm Al-Qura University, Makkah, SAU; 3 Faculty of Medicine, Department of Medicine, Umm Al-Qura University, Makkah, SAU

**Keywords:** saudi arabia, social media, university students, academic performance, lifestyle factors

## Abstract

Background and aim

A healthy lifestyle is defined as a way of living that reduces the likelihood of severe illness or early death. Factors required for a healthy lifestyle, such as regular physical activity, better sleep patterns, improved dietary habits, probable caffeine consumption, and decreased feelings of anxiety, are generally assumed to be important for high academic performance. This study aims to investigate the correlation between a healthy lifestyle and academic success among health profession students at Umm Al-Qura University, Saudi Arabia. By doing so, we could potentially lead to the implementation of targeted interventions to support students in achieving their best academic potential.

Methods

This is an observational cross-sectional study conducted among health profession students at Umm Al-Qura University. An online questionnaire was used to collect data on demographic information and the impact of lifestyle characteristics on academic performance from October to November 2023. Data were analyzed using RStudio (R version 4.3.1).

Results

A total of 652 students participated in the study. The majority were between the ages of 18 and 25 years (97.2%, n=634). Females constituted the majority of the participants (69.9%, n=456). Regarding the field of study, the College of Medicine had the highest representation (52.9%, n=345). Regarding body mass index, the normal weight category was the most prevalent, encompassing 59.8% (n=390) of the participants. The results show that the participants generally had a good grade point average (GPA) in the range of 3.50 to 4.00. Also, the time spent on social media applications was not correlated with academic performance (P=0.575). Importantly, the majority of participants perceived that lifestyle habits impacted their academic performance.

Conclusion

This study found that unhealthy lifestyle factors, such as lack of physical activity, inadequate sleep, poor dietary choices, smoking, and mental health issues such as anxiety, have a negative impact on academic performance. Therefore, the dissemination of relevant knowledge is needed to promote the importance of a healthy lifestyle and raise students' awareness.

## Introduction

A healthy lifestyle, as defined by the World Health Organization, is a manner of living that reduces the likelihood of severe illness or early death and offers more than just disease prevention. It promotes the overall well-being of the individual, including their physical, mental, and social health. Adopting a healthy lifestyle not only benefits the individual but also provides a positive example to those around them, such as family members, especially children [1]. The lifestyle factors of children and teenagers have a significant influence on their academic performance. For example, a lack of physical activity and unhealthy eating habits, such as irregular meal patterns and skipping breakfast, negatively affect their academic performance. Conversely, participation in a school breakfast program improves their academic performance and reduces absenteeism. Therefore, improving the dietary habits of students can benefit their academic outcomes [2].

Numerous other factors also impact students' academic success, including their motivation, physical well-being, and emotional state. In particular, elevated stress levels experienced by medical students can have adverse effects on their cognitive abilities and learning, resulting in lower academic performance. However, including physical activity in students' routines yields several advantages. It improves cognitive function, enhances learning abilities, boosts self-perception, increases arousal, reduces boredom, alleviates stress, stabilizes moods, promotes better sleep, and enhances attention span and concentration [3].

Sleep also plays a principal role in a healthy lifestyle. High-quality sleep leads to improved cognitive processing as a person gets healthier, which leads to excellent academic performance. On the other hand, a lack of sleep is associated with higher anxiety, depression, and stress [4]. A study conducted in 2020 in Saudi Arabia found no link between stress levels or sleep quality and academic performance. According to the study, stress was prevalent in 63.5% (n=179) of medical students, and 77% (n=217) reported poor sleep quality. Compared to students who did not have poor sleep quality, a large percentage of students with poor sleep quality expressed distress [5]. However, another study among medical students in Saudi Arabia discovered that academic performance was higher among students with poor sleep patterns than among those with better sleep patterns [4].

Studies examining this topic are limited, and to date, no study has evaluated the influence of lifestyle on the academic performance of health profession students at Umm Al-Qura University (UQU) in Saudi Arabia. Raising awareness of the importance of a healthy lifestyle and its impact on academic performance could improve student outcomes. Furthermore, the factors that affect students' academic performance and grade point average (GPA) should be known. Therefore, the primary goal of this study is to assess the impact of lifestyle factors, such as physical activity, sleep behavior, dietary habits, caffeine consumption, and anxiety, on the academic performance of health profession students at Umm Al-Qura University (UQU). The secondary goal is to aid in the development of programs that promote healthier lifestyles among college students, ultimately improving their academic outcomes and overall well-being.

## Materials and methods

Study design

The study was designed as an observational cross-sectional study. An online questionnaire was used to assess the influence of lifestyle factors on academic performance among health profession students at Umm Al-Qura University from October to November 2023. The study was conducted through a self-administered questionnaire distributed via online social media platforms. We collected the data and exported it to a Microsoft Excel (Microsoft Corporation, Redmond, Washington, United States) spreadsheet using Google Docs (Google LLC, Mountain View, California, United States) tools for data processing and analysis. The data were analyzed using RStudio (R version 4.3.1).

Sample size and sampling technique

The Raosoft Sample Size Calculator was used to determine the representative sample size required for this study: 370, with a 5% margin error and a 95% confidence level. The population was estimated at 8481. The study aimed to recruit more than the calculated sample size to overcome any exclusions. Therefore, convenience sampling techniques were employed.

Inclusion criteria and exclusion criteria

The entire population of students from Umm Al-Qura University Colleges of Medicine, Dentistry, Pharmacy, Nursing, Applied Medical Sciences, Public Health, and Health Informatics were eligible for inclusion in this study. Participation was voluntary. Health profession students from other universities and participants who did not complete the questionnaire were excluded from the study.

Data collection instruments and procedures

Data were collected using an electronic self-administered questionnaire that was adapted from a previous study on comparable objectives [6,7]. It included questions related to lifestyle factors such as physical activity, sleep behavior, dietary habits, caffeine consumption, social media usage, academic performance, and mental health issues like anxiety. The questionnaire was distributed electronically in the English language using Google Forms via different social media platforms (WhatsApp, Twitter, and Telegram).

Statical analysis

Statistical analysis was performed using RStudio (R version 4.3.1). Frequencies and percentages were used to express categorical variables. Factors associated with reporting negative effects of lifestyle factors on academic performance and having a low cumulative grade point average (GPA) of < 2.75 were assessed by constructing a univariable logistic regression analysis. The significantly associated variables from the univariable models were then incorporated into two multivariable models. The first model used the variable of negative effects of lifestyle factors on academic performance as a dependent variable, whereas the second variable employed having a low GPA as a dependent variable. The results of the regression analysis were expressed as odds ratios (ORs) and 95% confidence intervals (95% CIs). Statistical significance was deemed at p < 0.05.

Ethics and confidentiality

The questionnaire began with an explanation of the study's purpose and nature as well as a request for voluntary participation. The participant’s confidentiality was ensured by not collecting their names, numbers, or sensitive personal information. All information was kept private and used solely for scientific research. Ethical approval of the study was obtained before its initiation from the Biomedical Ethics Committee of Umm Al-Qura University (Approval No.: HAPO-02-K-012-2023-09-1772).

## Results

Initially, we collected data from 656 students. However, four records of students who disagreed with participating were excluded. Therefore, the analysis included a total of 652 records. The majority of the subjects in the current study were between the ages of 18 and 25 years, comprising 97.2% (n=634) of the sample. The largest proportion fell within the 21 to 25 age range (56.6%, n=369). Females constituted the majority of the participants, accounting for 69.9% (n=456) of the sample. Regarding the field of study, the College of Medicine had the highest representation (52.9%), n=345. Academic distribution showed that the fifth year had the highest frequency (24.7%, n=161) followed by the third year (19.5%, n=127). The majority of participants were single (95.6%, n=632). In terms of body mass index (BMI), the normal weight category was the most prevalent, encompassing 59.8% (n=390) of participants (Table 1).

**Table 1:** Demographic characteristics of participants BMI: body mass index

Characteristic	N (%)
Age	
18 to 20	265 (40.6%)
21 to 25	369 (56.6%)
26 to 30	15 (2.3%)
30 and above	3 (0.5%)
Gender	
Male	196 (30.1%)
Female	456 (69.9%)
Field of study	
College of Medicine	345 (52.9%)
College of Dentistry	14 (2.1%)
College of Pharmacy	135 (20.7%)
College of Nursing	45 (6.9%)
College of Applied Medical Sciences	68 (10.4%)
College of Public Health and Health Informatics	45 (6.9%)
Academic year	
1st year	76 (11.7%)
2nd year	122 (18.7%)
3rd year	127 (19.5%)
4th year	104 (16.0%)
5th year	161 (24.7%)
6th year	18 (2.8%)
7th year/Internship	44 (6.7%)
Marital status	
Single	623 (95.6%)
Married	23 (3.5%)
Divorced	6 (0.9%)
BMI	
Underweight	111 (17.0%)
Normal	390 (59.8%)
Overweight	123 (18.9%)
Obese	28 (4.3%)

The lifestyle characteristics of the participants reveal that almost one-quarter of the sample does not engage in physical activity regularly (23.0%, n=150) while 28.5% (n=186) reported engagement less than once a week. A notable proportion, comprising 34.7% (n=226), reported spending no time on exercise per day. Sleep patterns indicate that the majority (38.3%, n=250) habitually go to sleep between 1 a.m. and 2 a.m. A significant number of participants (37.6%, n=245) reported getting five to six hours of sleep per night. In terms of dietary habits, 49.4% (n=322) reported consuming two meals per day, and 33.6% (n=219) reported having three meals per day. Additionally, 33.9% (n=221) reported consuming fast food two times per week. Furthermore, 50.5% (n=329) reported drinking one to two cups of coffee per day, and 39.7% (n=259) reported never consuming tea. A noteworthy finding is that 64.9% (n=423) of participants spend more than four hours per day using a computer, cellphone, or the internet for pleasure. Additionally, 17.9% (n=117) of students had ever been diagnosed with a generalized anxiety disorder. More details about lifestyle characteristics are listed in Table 2.

**Table 2:** Lifestyle characteristics of participants

Characteristic	N (%)
How often do you engage in physical activity per week?	
Never	150 (23.0%)
< once a week	186 (28.5%)
1 to 3 times a week	174 (26.7%)
3 to 4 times a week	98 (15.0%)
> 4 times a week	44 (6.7%)
How many minutes of exercise per day?	
Never	226 (34.7%)
15 to 30 minutes	213 (32.7%)
31 to 60 minutes	138 (21.2%)
61 to 120 minutes	60 (9.2%)
> 120 minutes	15 (2.3%)
On average, what time do you usually go to sleep?	
9 to 10 PM	98 (15.0%)
11 PM to 12 AM	182 (27.9%)
1 to 2 AM	250 (38.3%)
3 to 4 AM	99 (15.2%)
5 to 6 AM	23 (3.5%)
How many hours of sleep do you usually get per night?	
< 5 h	110 (16.9%)
5 to 6 h	245 (37.6%)
7 to 8 h	183 (28.1%)
9 to 10 h	80 (12.3%)
> 10 h	34 (5.2%)
Number of meals per day?	
1 meal	64 (9.8%)
2 meals	322 (49.4%)
3 meals	219 (33.6%)
4 to 5 meals	42 (6.4%)
> 5 meals	5 (0.8%)
Do you have breakfast every day?	
Never	61 (9.4%)
Rarely	140 (21.5%)
Sometimes	210 (32.2%)
Often	101 (15.5%)
Always	140 (21.5%)
Do you have a recommended daily amount of fruits and vegetables?	
Never	115 (17.6%)
Rarely	210 (32.2%)
Sometimes	221 (33.9%)
Often	75 (11.5%)
Always	31 (4.8%)
How often do you consume fast food per week?	
1 time	145 (22.2%)
2 times	221 (33.9%)
3 to 6 times	219 (33.6%)
7 to 9 times	44 (6.7%)
> 9 times	23 (3.5%)
How many cups of coffee per day?	
Never	200 (30.7%)
1 to 2 cups	329 (50.5%)
3 to 4 cups	83 (12.7%)
5 to 6 cups	23 (3.5%)
> 6 cups	17 (2.6%)
How many cups of tea per day?	
Never	259 (39.7%)
1 to 2 cups	295 (45.2%)
3 to 4 cups	70 (10.7%)
5 to 6 cups	13 (2.0%)
> 6 cups	15 (2.3%)
How many cigarettes do you smoke per day?	
Never	600 (92.0%)
1 to 5 cigarettes	26 (4.0%)
6 to 10 cigarettes	14 (2.1%)
11 to 15 cigarettes	4 (0.6%)
> 15 cigarettes	8 (1.2%)
How many hours are spent on using a computer, cellphone, or internet for pleasure per day?	
Never	23 (3.5%)
1/2 hour	28 (4.3%)
1 to 2 hours	48 (7.4%)
3 to 4 hours	130 (19.9%)
> 4 hours	423 (64.9%)
How many hours are spent watching TV, movies, or playing video games per day?	
Never	154 (23.6%)
1/2 hour	201 (30.8%)
1 to 2 hours	133 (20.4%)
3 to 4 hours	84 (12.9%)
> 4 hours	80 (12.3%)
Have you ever been diagnosed with generalized anxiety disorder?	117 (17.9%)

**Figure 1:**
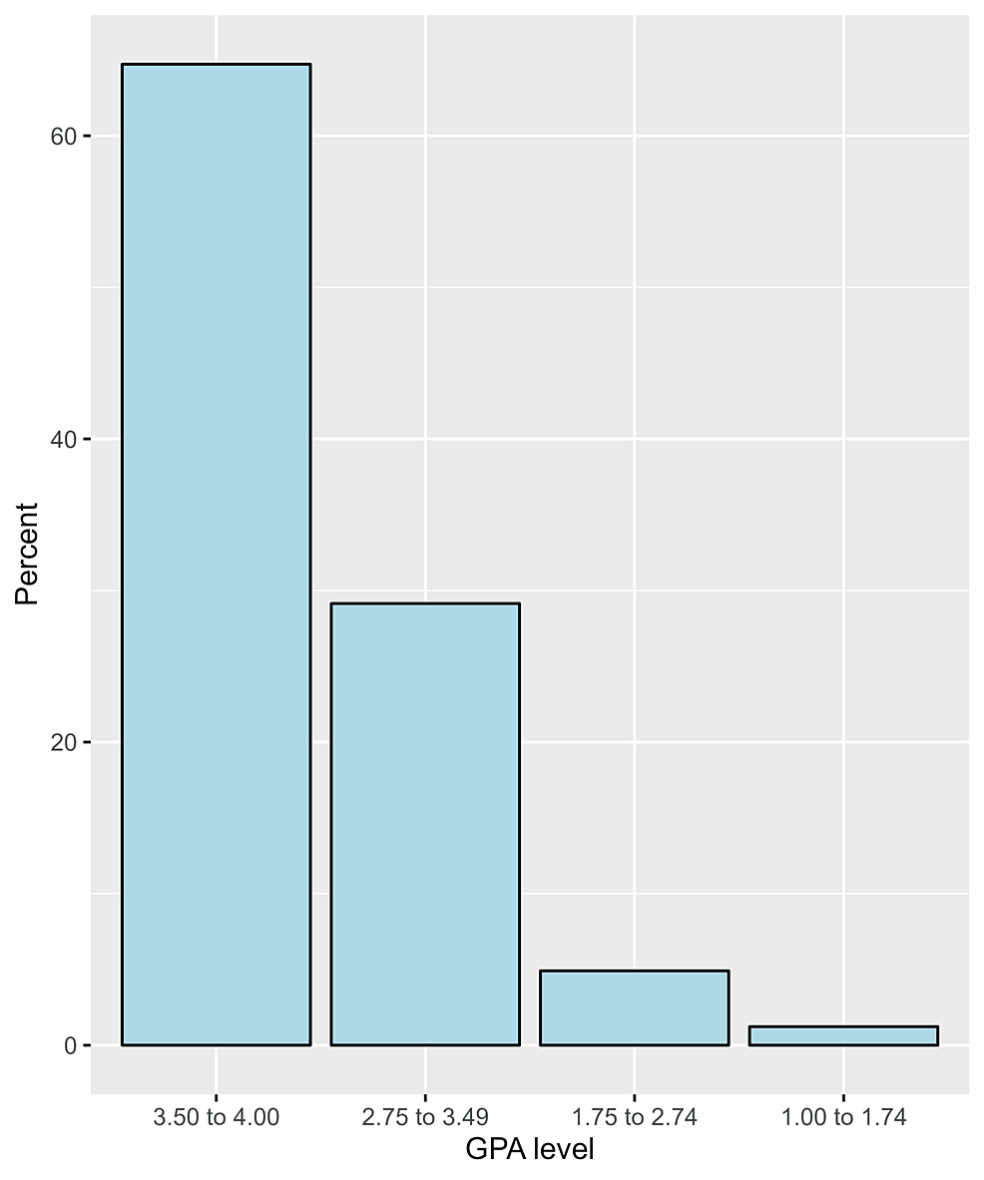
The proportions of participants’ responses regarding their current cumulative grade point average (GPA)

**Figure 2:**
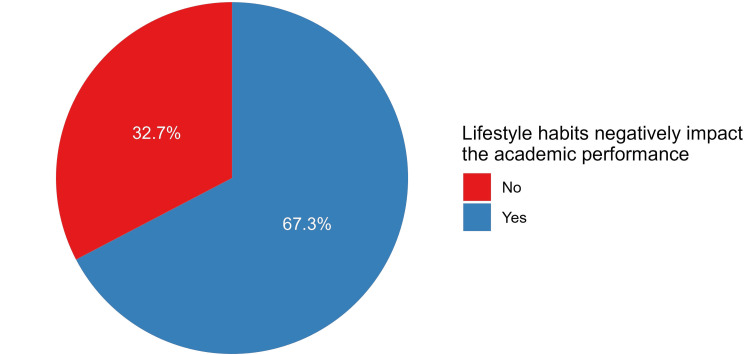
The proportions of participants’ responses regarding the negative impact of lifestyle characteristics on academic performance

The academic achievement characteristics of the participants indicate that a majority, comprising 64.7% (n=422), have a current cumulative grade point average (GPA) in the range of 3.50 to 4.00 (Figure 1). Regarding class attendance, 59.8% (n=390) reported not missing any classes per month due to health issues. The majority of participants (82.5%, n=538) did not miss any exams in the past academic year due to health issues. In terms of study hours per week, a significant portion (29.3%, n=191) reported studying less than 10 hours per week on average (Table 3). Importantly, 67.3% (n=439) of participants stated that lifestyle habits negatively impact their academic performance (Figure 2).

**Table 3:** Characteristics of the academic achievement

Characteristic	N (%)
What is the number of classes missed per month on average due to health issues?	
None	390 (59.8%)
1 to 2 classes	197 (30.2%)
3 to 4 classes	48 (7.4%)
> 4 classes	17 (2.6%)
How many hours did you study per week on average?	
< 10 h	191 (29.3%)
10 to 15 h	189 (29.0%)
16 to 20 h	125 (19.2%)
21 to 25 h	85 (13.0%)
> 25 h	62 (9.5%)
What is the number of missed exams in the past academic year due to health issues?	
None	538 (82.5%)
1 to 2 exams	83 (12.7%)
3 to 4 exams	23 (3.5%)
> 4 exams	8 (1.2%)

Overweight students were more likely to report negative effects of lifestyle factors on their academic performance (OR=2.18, 95% CI=1.10 to 4.38, p=0.027). Additionally, students who reported having breakfast often or always exhibited significantly lower odds of experiencing negative academic effects (often: OR=0.36, 95% CI=0.14 to 0.88, p=0.030; always: OR=0.31, 95% CI=0.12 to 0.73, p=0.010). Moreover, the frequency of consuming fast food per week showed a significant association, with an increase in the odds of negative academic effects as the frequency of fast food consumption rose (two times: OR=1.88, 95% CI=1.13 to 3.13, p=0.015; three to six times: OR=3.18, 95% CI=1.89 to 5.40, p<0.001). Students who used to smoke one to five cigarettes per day were more likely to experience negative academic effects (OR=10.0, 95% CI=1.83 to 190, p=0.032). Lastly, students ever diagnosed with generalized anxiety disorder were at a significantly higher risk of negative academic outcomes (OR=3.94, 95% CI=2.10 to 7.86, p<0.001) (Table 4).

**Table 4:** Regression analysis of the risk factors for negative effects on academic performance OR: odds ratio; CI: confidence interval; BMI: body mass index

	Univariable				Multivariable		
Characteristic	N	OR	95% CI	p-value	OR	95% CI	p-value
Age	652						
18 to 20		Reference	Reference				
21 to 25		1.30	0.93, 1.82	0.121			
26 to 30		1.14	0.39, 3.74	0.821			
30 and above		NA	NA	0.978			
Gender	652						
Male		Reference	Reference				
Female		0.84	0.59, 1.21	0.360			
Field of study	652						
College of Medicine		Reference	Reference		Reference	Reference	
College of Dentistry		0.88	0.30, 2.91	0.817	0.99	0.28, 3.72	0.983
College of Pharmacy		1.57	1.00, 2.50	0.053	1.68	1.00, 2.87	0.053
College of Nursing		1.34	0.68, 2.79	0.412	2.20	1.01, 5.06	0.053
College of Applied Medical Sciences		0.66	0.39, 1.12	0.118	0.73	0.39, 1.35	0.307
College of Public Health and Health Informatics		0.51	0.27, 0.96	0.035	0.54	0.26, 1.12	0.097
Academic year	652						
1st year		Reference	Reference				
2nd year		0.82	0.44, 1.50	0.518			
3rd year		0.79	0.43, 1.43	0.433			
4th year		1.19	0.62, 2.28	0.591			
5th year		1.23	0.67, 2.21	0.500			
6th year		0.37	0.13, 1.05	0.062			
7th year/Internship		0.89	0.41, 1.99	0.777			
Marital status	652						
Single		Reference	Reference				
Married		1.77	0.69, 5.41	0.268			
Divorced		0.49	0.09, 2.67	0.385			
BMI	652						
Underweight		Reference	Reference		Reference	Reference	
Normal		0.74	0.47, 1.16	0.199	0.82	0.48, 1.37	0.445
Overweight		2.00	1.10, 3.70	0.024	2.18	1.10, 4.38	0.027
Obese		1.69	0.66, 4.91	0.298	2.14	0.72, 7.05	0.187
How often do you engage in physical activity per week?	652						
Never		Reference	Reference		Reference	Reference	
< once a week		0.66	0.40, 1.06	0.090	0.87	0.46, 1.65	0.673
1 to 3 times a week		0.68	0.41, 1.11	0.122	0.93	0.45, 1.93	0.841
3 to 4 times a week		0.44	0.25, 0.77	0.004	0.78	0.34, 1.79	0.561
> 4 times a week		0.23	0.11, 0.47	<0.001	0.40	0.15, 1.07	0.069
How many minutes of exercise per day?	652						
Never		Reference	Reference		Reference	Reference	
15 to 30 minutes		0.62	0.41, 0.93	0.023	0.71	0.39, 1.25	0.237
31 to 60 minutes		0.61	0.39, 0.97	0.037	0.77	0.39, 1.52	0.443
61 to 120 minutes		0.41	0.23, 0.75	0.003	0.62	0.26, 1.49	0.286
> 120 minutes		0.67	0.23, 2.24	0.489	0.61	0.15, 2.75	0.496
On average, what time do you usually go to sleep?	652						
9 to 10 PM		Reference	Reference				
11 PM to 12 AM		0.76	0.45, 1.26	0.287			
1 to 2 AM		1.39	0.84, 2.29	0.193			
3 to 4 AM		1.42	0.77, 2.61	0.261			
5 to 6 AM		1.00	0.39, 2.69	0.994			
How many hours of sleep do you usually get per night?	652						
< 5 h		Reference	Reference		Reference	Reference	
5 to 6 h		0.55	0.33, 0.92	0.025	0.69	0.37, 1.25	0.227
7 to 8 h		0.56	0.32, 0.95	0.036	0.92	0.49, 1.72	0.800
9 to 10 h		0.55	0.29, 1.03	0.064	0.61	0.28, 1.30	0.199
> 10 h		0.54	0.24, 1.26	0.146	0.47	0.18, 1.24	0.124
Number of meals per day?	652						
1 meal		Reference	Reference				
2 meals		0.89	0.48, 1.57	0.682			
3 meals		0.76	0.41, 1.38	0.380			
4 to 5 meals		1.35	0.56, 3.38	0.507			
> 5 meals		0.63	0.10, 5.10	0.632			
Do you have breakfast every day?	652						
Never		Reference	Reference		Reference	Reference	
Rarely		0.39	0.17, 0.83	0.020	0.42	0.16, 1.00	0.058
Sometimes		0.44	0.19, 0.92	0.038	0.58	0.23, 1.35	0.225
Often		0.26	0.11, 0.57	0.001	0.36	0.14, 0.88	0.030
Always		0.22	0.09, 0.46	<0.001	0.31	0.12, 0.73	0.010
Do you have a recommended daily amount of fruits and vegetables?	652						
Never		Reference	Reference		Reference	Reference	
Rarely		1.11	0.66, 1.85	0.680	1.47	0.81, 2.68	0.204
Sometimes		0.63	0.38, 1.02	0.063	0.84	0.46, 1.53	0.575
Often		0.49	0.26, 0.90	0.023	0.67	0.32, 1.40	0.285
Always		0.81	0.35, 1.97	0.629	1.49	0.54, 4.26	0.446
How often do you consume fast food per week?	652						
1 time		Reference	Reference		Reference	Reference	
2 times		2.08	1.36, 3.21	<0.001	1.88	1.13, 3.13	0.015
3 to 6 times		3.33	2.13, 5.27	<0.001	3.18	1.89, 5.40	<0.001
7 to 9 times		2.96	1.43, 6.55	0.005	1.79	0.76, 4.40	0.191
> 9 times		1.53	0.63, 3.89	0.350	0.80	0.28, 2.36	0.678
How many cups of coffee per day?	652						
Never		Reference	Reference				
1 to 2 cups		0.78	0.54, 1.14	0.206			
3 to 4 cups		1.14	0.66, 2.04	0.640			
5 to 6 cups		1.00	0.41, 2.72	0.995			
> 6 cups		1.43	0.48, 5.22	0.549			
How many cups of tea per day?	652						
Never		Reference	Reference				
1 to 2 cups		1.22	0.86, 1.73	0.272			
3 to 4 cups		1.65	0.92, 3.04	0.100			
5 to 6 cups		3.13	0.82, 20.5	0.143			
> 6 cups		1.14	0.39, 3.75	0.817			
How many cigarettes do you smoke per day?	652						
Never		Reference	Reference		Reference	Reference	
1 to 5 cigarettes		13.2	2.76, 236	0.012	10.0	1.83, 190	0.032
6 to 10 cigarettes		3.16	0.85, 20.4	0.134	2.38	0.51, 17.6	0.317
11 to 15 cigarettes		NA	NA, NA	0.975	NA	NA, NA	0.982
> 15 cigarettes		0.88	0.21, 4.31	0.859	0.94	0.19, 5.36	0.945
How many hours are spent using a computer, cellphone, or internet for pleasure per day?	652						
Never		Reference	Reference				
1/2 hour		1.62	0.42, 6.50	0.479			
1 to 2 hours		0.95	0.29, 2.87	0.929			
3 to 4 hours		0.48	0.16, 1.24	0.149			
> 4 hours		0.76	0.27, 1.88	0.575			
How many hours are spent watching TV, movies, or playing video games per day?	652						
Never		Reference	Reference				
1/2 hour		1.00	0.64, 1.54	0.983			
1 to 2 hours		1.29	0.79, 2.13	0.312			
3 to 4 hours		1.17	0.67, 2.08	0.580			
> 4 hours		1.78	0.98, 3.35	0.064			
Have you ever been diagnosed with generalized anxiety disorder?	652						
No		Reference	Reference		Reference	Reference	
Yes		4.00	2.33, 7.33	<0.001	3.94	2.10, 7.86	<0.001

In the multivariable regression analysis aimed at identifying risk factors for obtaining a low GPA (< 2.75), several variables exhibited significant associations. Notably, sleep duration emerged as a significant factor, with students sleeping for more than 10 hours per night at a higher risk of achieving a low GPA (OR=4.60, 95% CI=1.12 to 19.6, p=0.033). Conversely, students who reported having two meals and three meals a day had a significantly lower risk of obtaining a low GPA compared to those with one meal (OR=0.36, 95% CI=0.14 to 0.94, p=0.031 and OR=0.31, 95% CI=0.10 to 0.94, p=0.036, respectively). Moreover, students ever diagnosed with generalized anxiety disorder were more likely to achieve a low GPA (OR=2.27, 95% CI=1.00 to 4.91, p=0.042). The number of cigarettes smoked per day was also a significant factor, with students smoking six to 10 cigarettes per day having a higher risk of a low GPA (OR=5.57, 95% CI=1.26 to 20.9, p=0.014) (Table 5).

**Table 5:** Regression analysis of the risk factors for obtaining a low GPA (< 2.75) OR: odds ratio; CI: confidence interval; BMI: body mass index

	Univariable				Multivariable		
Characteristic	N	OR	95% CI	p-value	OR	95% CI	p-value
Age	652						
18 to 20		Reference	Reference		Reference	Reference	
21 to 25		1.40	0.70, 2.96	0.356	1.22	0.57, 2.71	0.619
26 to 30		7.67	1.91, 26.4	0.002	4.07	0.87, 16.7	0.059
30 and above		10.5	0.47, 118	0.062	0.74	0.02, 14.2	0.841
Gender	652						
Male		Reference	Reference				
Female		0.89	0.46, 1.81	0.729			
Field of study	652						
College of Medicine		Reference	Reference				
College of Dentistry		2.33	0.35, 9.25	0.286			
College of Pharmacy		0.77	0.30, 1.74	0.548			
College of Nursing		1.00	0.23, 3.03	>0.999			
College of Applied Medical Sciences		0.88	0.25, 2.37	0.811			
College of Public Health and Health Informatics		0.32	0.02, 1.57	0.268			
Academic year	652						
1st year		Reference	Reference				
2nd year		0.86	0.27, 3.02	0.809			
3rd year		0.46	0.11, 1.80	0.261			
4th year		1.35	0.44, 4.54	0.609			
5th year		1.04	0.36, 3.41	0.942			
6th year		NA	NA	0.987			
7th year/Internship		1.42	0.33, 5.66	0.616			
Marital status	652						
Single		Reference	Reference				
Married		0.70	0.04, 3.48	0.730			
Divorced		3.08	0.16, 19.7	0.310			
BMI	652						
Underweight		Reference	Reference				
Normal		0.88	0.40, 2.14	0.765			
Overweight		0.43	0.11, 1.42	0.182			
Obese		1.55	0.32, 5.78	0.542			
How often do you engage in physical activity per week?	652						
Never		Reference	Reference				
< once a week		0.45	0.16, 1.15	0.102			
1 to 3 times a week		1.16	0.53, 2.60	0.703			
3 to 4 times a week		0.36	0.08, 1.18	0.124			
> 4 times a week		0.55	0.08, 2.11	0.442			
How many minutes of exercise per day?	652						
Never		Reference	Reference				
15 to 30 minutes		0.98	0.43, 2.21	0.957			
31 to 60 minutes		1.14	0.46, 2.72	0.765			
61 to 120 minutes		1.49	0.46, 4.14	0.467			
> 120 minutes		1.17	0.06, 6.57	0.884			
On an average, what time do you usually go to sleep?	652						
9 to 10 PM		Reference	Reference				
11 PM to 12 AM		1.28	0.49, 3.71	0.627			
1 to 2 AM		0.91	0.35, 2.63	0.851			
3 to 4 AM		0.65	0.16, 2.33	0.508			
5 to 6 AM		1.46	0.20, 6.87	0.657			
How many hours of sleep do you usually get per night?	652						
< 5 h		Reference	Reference		Reference	Reference	
5 to 6 h		0.89	0.31, 2.93	0.841	1.52	0.48, 5.49	0.491
7 to 8 h		1.21	0.42, 3.99	0.730	1.91	0.60, 6.97	0.295
9 to 10 h		2.66	0.88, 8.97	0.091	3.47	1.03, 13.1	0.051
> 10 h		4.50	1.27, 16.7	0.019	4.60	1.12, 19.6	0.033
Number of meals per day?	652						
1 meal		Reference	Reference		Reference	Reference	
2 meals		0.30	0.13, 0.71	0.005	0.36	0.14, 0.94	0.031
3 meals		0.26	0.10, 0.66	0.004	0.31	0.10, 0.94	0.036
4 to 5 meals		0.27	0.04, 1.10	0.103	0.31	0.04, 1.41	0.167
> 5 meals		1.35	0.07, 10.4	0.798	0.79	0.03, 7.41	0.854
Do you have breakfast every day?	652						
Never		Reference	Reference				
Rarely		0.59	0.18, 2.06	0.384			
Sometimes		0.80	0.29, 2.56	0.681			
Often		0.58	0.16, 2.18	0.410			
Always		0.77	0.25, 2.60	0.651			
Do you have a recommended daily amount of fruits and vegetables?	652						
Never		Reference	Reference				
Rarely		0.53	0.20, 1.39	0.188			
Sometimes		0.68	0.28, 1.70	0.392			
Often		0.66	0.17, 2.12	0.508			
Always		2.83	0.88, 8.59	0.069			
How often do you consume fast food per week?	652						
1 time		Reference	Reference				
2 times		1.21	0.45, 3.59	0.709			
3 to 6 times		1.95	0.79, 5.52	0.171			
7 to 9 times		1.70	0.35, 6.73	0.469			
> 9 times		3.47	0.69, 14.3	0.095			
How many cups of coffee per day?	652						
Never		Reference	Reference				
1 to 2 cups		0.74	0.35, 1.59	0.424			
3 to 4 cups		1.53	0.59, 3.79	0.362			
5 to 6 cups		2.16	0.47, 7.41	0.260			
> 6 cups		NA	NA	0.988			
How many cups of tea per day?	652						
Never		Reference	Reference		Reference	Reference	
1 to 2 cups		1.01	0.47, 2.20	0.972	1.12	0.50, 2.55	0.777
3 to 4 cups		2.10	0.76, 5.36	0.129	2.18	0.73, 6.02	0.141
5 to 6 cups		3.44	0.50, 14.6	0.132	1.31	0.14, 8.37	0.793
> 6 cups		4.73	0.99, 17.3	0.028	3.57	0.62, 15.5	0.111
How many cigarettes do you smoke per day?	652						
Never		Reference	Reference		Reference	Reference	
1 to 5 cigarettes		0.69	0.04, 3.40	0.717	0.48	0.03, 2.66	0.494
6 to 10 cigarettes		6.87	1.81, 21.8	0.002	5.57	1.26, 20.9	0.014
11 to 15 cigarettes		5.73	0.28, 46.1	0.135	1.62	0.06, 23.9	0.738
> 15 cigarettes		2.45	0.13, 14.4	0.407	1.19	0.05, 10.3	0.895
How many hours are spent using a computer, cellphone, or internet for pleasure per day?	652						
Never		Reference	Reference				
1/2 hour		1.11	0.22, 6.20	0.898			
1 to 2 hours		0.78	0.17, 4.08	0.744			
3 to 4 hours		0.27	0.06, 1.38	0.086			
> 4 hours		0.38	0.12, 1.71	0.143			
How many hours are spent watching TV, movies, or playing video games per day?	652						
Never		Reference	Reference				
1/2 hour		0.87	0.31, 2.53	0.793			
1 to 2 hours		2.47	0.99, 6.70	0.059			
3 to 4 hours		1.62	0.50, 5.03	0.403			
> 4 hours		1.40	0.40, 4.53	0.577			
Have you ever been diagnosed with generalized anxiety disorder?	652						
No		Reference	Reference		Reference	Reference	
Yes		3.00	1.50, 5.83	0.001	2.27	1.00, 4.91	0.042

## Discussion

This study aimed to determine the association between lifestyle habits and the academic performance of students in the healthcare profession, with a focus on body weight, eating habits, smoking, sleep patterns, and mental health. The majority of the study participants were female, accounting for 69.9% (n=456) of the sample, and the highest number of responses (52.9%, n=345) were from the College of Medicine. Most of the participants (59.8%, n=390) were of average weight (Table 1).

Previous studies have demonstrated that obesity and being overweight can negatively impact students' concentration, leading to lower academic performance [8-11]. These results are consistent with the current study's results, which show that overweight students are highly likely to report a negative effect of lifestyle factors on their academic performance. However, numerous studies suggest that the relationship between body weight and academic performance is far more complex and involves other contributing factors, such as gender, socioeconomic status, and mental health [12,13]. On the other hand, some studies have found no relation between body mass index (BMI) and academic performance [14,15].

Eating habits have a significant impact on academic performance. As many studies have shown, students who have regular nutritious meals tend to have better concentration, memory, and academic performance than those with poor eating habits such as skipping meals and consuming high amounts of fast food [16-19]. This is in line with the current study's results, which found that students who often or always have breakfast are less likely to experience low academic performance. Moreover, students who have two to three meals a day have a significantly lower risk of obtaining a low grade point average (GPA) compared to those who have one meal a day. Furthermore, the consumption of fast food was significantly correlated with poor academic performance.

The study results also show that students who smoke one to five cigarettes per day are more likely to experience low academic performance. Additionally, the number of cigarettes smoked per day can be an important factor in determining academic performance. This study found that students who smoke six to 10 cigarettes per day are at higher risk of obtaining low GPAs. This finding is supported by a recent study conducted in Saudi Arabia that examined the impact of smoking and nicotine dependence on the academic performance of health sciences students. Heavy smokers were found to have a lower grade point average (GPA) and a higher absenteeism rate than light smokers [20].

This study identified a prevalent disruption in the sleep patterns of students, with a significant number consistently sleeping between 1 a.m. and 2 a.m. and obtaining only five to six hours of sleep per night. This raises concerns about inadequate sleep hygiene; existing literature links insufficient sleep and poor sleeping habits to adverse academic outcomes [21,22]. Surprisingly, this study's findings revealed that students who sleep over 10 hours per night face a higher risk of achieving a lower grade point average (GPA), suggesting that both inadequate and excessive sleep might negatively impact academic performance. Therefore, it is crucial to address these issues through targeted interventions aimed at improving sleep habits among students. 

Additionally, this study showed the substantial effects of mental health on academic performance, particularly among students diagnosed with generalized anxiety disorder. These students exhibited a significantly higher risk of low academic performance and achieving a lower grade point average (GPA). This aligns with the findings of previous studies among both medical and non-medical university students: higher anxiety levels are linked to lower grades and poor academic performance [23,24]. This emphasizes the importance of robust student support systems in universities, such as counseling services and stress management programs. The implementation of these measures could significantly reduce the adverse impact of mental health issues on students' academic performance.

This study has a few limitations. First, a cross-sectional study design that provides knowledge for a specific point in time with a convenient sampling method might not be the ideal sampling strategy for a large study setting involving both genders. In addition, responses could be the source of bias because students who underreport their emotions, and students' perceptions of survey questions may vary and affect the accuracy of their responses. The results of this study might not be applicable elsewhere in the world due to differences in social, demographic, and cultural values.

## Conclusions

This study found that unhealthy lifestyle factors, such as lack of physical activity, inadequate sleep, poor dietary choices, smoking, and mental health issues such as anxiety, have a negative impact on academic performance. Therefore, the dissemination of relevant knowledge is needed to promote the importance of a healthy lifestyle and raise students' awareness.
